# A Self-Priming Microfluidic Chip with Cushion Chambers for Easy Digital PCR

**DOI:** 10.3390/bios11050158

**Published:** 2021-05-18

**Authors:** Gangwei Xu, Huaqing Si, Fengxiang Jing, Peng Sun, Dongping Wu

**Affiliations:** 1 State Key Laboratory of ASIC and System, School of Microelectronics, Fudan University, Shanghai 200433, China; 18112020050@fudan.edu.cn (G.X.); 17112020035@fudan.edu.cn (H.S.); 18112020068@fudan.edu.cn (P.S.); 2 Shanghai Turtle Technology Company Limited, Shanghai 200439, China; fengxiangjing@turtle-tech.cn

**Keywords:** microfluidic chip, self-priming, cushion chambers, digital PCR

## Abstract

A polydimethylsiloxane (PDMS)-based self-priming microfluidic chip with cushion chambers is presented in this study for robust and easy-operation digital polymerase chain reaction (dPCR). The chip has only one inlet and can partition samples autonomously through negative pressure, provided by a de-gassed PDMS layer with a multi-level vertical branching microchannel design. Meanwhile, cushion chambers make the chip capable of very robust use for sample partitioning. Finally, the proposed microfluidic chip showed excellent performance in the absolute quantification of a target gene by performing quantitative detection of a 10-fold serial dilution DNA template. Owing to its characteristics of easy operation, low cost, and high robustness, the proposed dPCR chip is expected to further promote the extensive application of digital PCR, especially in resource-limited settings.

## 1. Introduction

Compared with real-time quantitative polymerase chain reaction (qPCR), digital polymerase chain reaction (dPCR) is a technique of absolute nucleic acid quantification with higher sensitivity and accuracy. In dPCR, the reagents are divided into thousands, or even millions, of separate partitions. After PCR amplification, positive partitions are counted and the absolute concentration of target DNA can be directly obtained according to the Poisson statistics [1]. DPCR is fast becoming a powerful tool for diagnosing many kinds of cancer, such as lung [2], colorectal [3], and breast cancers [4,5], owing to its high precision and sensitivity for DNA quantification.

The recent development of dPCR platforms has been supported by microfluidics, i.e., the miniaturization of fluid handling, and can be broadly classified into two categories based on partition format: liquid droplets dPCR (ddPCR) [6,7,8,9] and physical partition dPCR (pdPCR) [10,11]. Most of these methods allow for simple reagent compartmentalization. In ddPCR platforms, droplets are monodispersed and free moving, which may cause collisions and coalescence during experimental processes, and thus affect the accuracy of the quantitative results [12,13,14]. On the contrary, pdPCR platforms, in which the reagent is partitioned in isolated chambers or microwells of a fixed structure of equal volume [15], have a higher uniformity of partitions and avoid the coalescence of droplets [16]. In addition, pdPCR makes it easier to achieve visual amplification results in real time under a fluorescence microscope.

The previously developed pdPCR platforms usually need additional pump devices or complex micro-valve structures to achieve sample dispersion. For example, the BioMark system is a typical representative that requires a large array of micro-valves to realize the partitioning of samples [17]. For other major microfluidic pdPCR studies, such as microwell chips [18,19] and self-digitization chips [16,20,21], an oil phase was used instead of a micro-valve to seal the aqueous solution for realizing the separation of samples. However, the microwell chip separates samples by the difference in hydrophilicity between the inner surface of the microwells and the top surface of the chip [18], and the self-digitization chip divides samples using an external polydimethylsiloxane (PDMS) pump [22]. These chips have a reagent outlet, and thus have a potential risk of cross-contamination. SlipChip, a typical pdPCR platform, does not require pumping or valving and is relatively easy operate with effective partitioning, however, it needs precision alignment during reagent partitioning [23,24]. A chip fabricated by PDMS with a fractal branching microchannel net can realize self-digitization with only one inlet [11]. However, it requires a very high uniformity of suction distribution. Once there is uneven pressure distribution, samples cannot be separated successfully.

To solve the aforementioned problem, a multi-level vertical microchannel microfluidic chip with cushion chambers is proposed in this study for robust and easy dPCR. Cushion chambers are introduced at each end of a vertical branching microchannel, which greatly improve the tolerance of the dPCR chip. The self-partitioning results of the developed chip were verified using red dye, and the buffer effect of the chip’s cushion chambers was verified with fluorescence dye. With cushion chambers and a multi-level vertical branching microchannel, the proposed microfluidic chip can actively realize reagent compartmentalization without an additional pump and valve. Owing to its advantages of a simple manufacturing process, low production cost, and easy operation processes, the proposed chip can further improve the application range and reliability of dPCR.

## 2. Experimental

### 2.1. Chip Design and Fabrication

**Figure 1:**
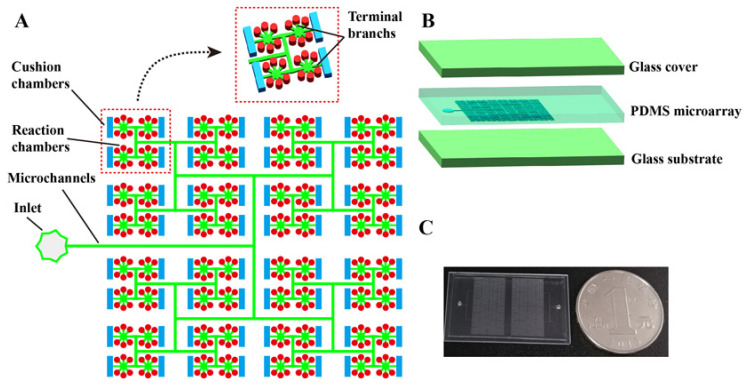
Schematic of self-priming dPCR chip with cushion chambers. (**A**) Schematic of the dPCR chip structural design. The chip contains two types of chambers with different volumes and shapes. The chip has only one inlet, without any outlets. The main channel is divided vertically into many branches, step by step. The end of each branch is connected with six reaction chambers and one cushion chamber. (**B**) Schematic diagram of the layered structure of the dPCR chip, which is composed of two layers of glass coverslips and the PDMS (inlet and microarray-layer) sandwiched between them. (**C**) Photograph of the prototype dPCR chip. The chip contains two panels, and each panel has 3072 reaction chambers and 512 cushion chambers. The size of the chip is 20 mm × 35 mm.

As shown in Figure 1A,B, the proposed dPCR chip has a glass–PDMS–glass multi-layer structure. The PDMS micro-array layer in this chip acts as the structural layer, and the cover glass and substrate glass layers provide a robust seal and support for the structural layer and reduce water loss during the thermal cycle. Unlike traditional dPCR chips, the micro-array layer of the chip contains two types of chambers with different volumes and shapes, in which the cylindrical chambers act as “reaction chambers” and the rectangular chambers act as “cushion chambers”. The main channel is divided vertically step by step into many branches, as shown in Figure 1A. At the end of each branch, there are six cylindrical chambers and one rectangular chamber. All chambers are 100 μm high, the diameter of the cylindrical chambers is 100 μm, and the width and length of the rectangular chamber are 100 and 400 μm, respectively. The height of the channels is 30 μm and the width of the main channel is 80 μm. The terminal branch (shown in Figure 1A) connected with the cylindrical chamber is trapezoid shaped, the top and bottom edges of which are 50 and 80 μm, respectively. The terminal branch connected with the rectangular chamber is rectangular, and the width of that is 50 μm. The proposed microfluidic chip was fabricated using the multi-layer soft lithography process described in detail in our previous work [25]. In addition, the chip is scalable, due to its symmetrical structure. As shown in Figure S1, when n equals 10, the total number of the chip can reach more than 20,000. In the actual preparation, the PDMS micro-array layer contains two panels, and each panel has 3072 reaction chambers and 512 cushion chambers. A prototype chip, of size 20 mm × 35 mm, is shown in Figure 1C.

### 2.2. Chip Operation

As shown in Figure S2 of the Supplementary Material, the operating procedure of the proposed chip can be briefly described as follows: First, the chip inlet was sealed by a piece of transparent adhesive-tape. Next, to evacuate the air dissolved in the PDMS layer, the chip was degassed to 0.1 kPa for 20 min in a vacuum chamber (Figure S2A). Meanwhile, the reaction reagent and pre-mixed oil were prepared. The chip was taken out after de-gassing and used within 20 min. Then, a two-level micro-pipette was used to successively draw 10 μL of pre-mixed oil (part A:part B = 3:1, obtained from Saint Gene Technologies Co Ltd., Suzhou, China) and the PCR pre-mix. After the tape was torn off, the PCR pre-mix and pre-mixed oil was added into the inlet of the chip immediately. The reagent was drawn quickly and completely into the chip in approximately 20 s and the pre-mixed oil was then drawn into the channel of the chip following the reagent (Figure S2B). After approximately 2 min, the reagent was completely drawn into each chamber and separated by oil. Finally, a glass cover slip was placed on the top surface of the chip coated with 10 μL of pre-mixed oil (Figure S2C). The chip was transferred to an in situ PCR instrument (Turtle Tech Ltd., Shanghai, China) for thermal cycling.

### 2.3. PCR Conditions

In this work, KRAS wild DNA templates were used to evaluate the performance of the proposed microfluidic chip. All DNA templates were extracted according to the manufacturer’s protocol provided in the corresponding kits. All DNA samples and the reaction mix were stored at −20 °C prior to use. To test the linearity of the chip, the prepared KRAS wild DNA templates were measured by a commercial digital PCR platform (BioDigital dPCR System, Turtle Tech. Ltd., Shanghai, China) and then diluted into a series of dilutions spanning 4 orders of magnitude.

The dPCR pre-mix (every 10 μL) consisted of 10 × BioDigital dPCR Mix 1 μL, forward/reverse primers, and probes with a concentration and volume of 0.4 mM and 0.5 μL, respectively, and a serially diluted template 1 μL, the rest of the volume (4 µL) comprised nuclease-free water. All primers and probes are presented in Table S2. After sampling, the dPCR chip was transferred to an in situ PCR instrument for PCR thermal cycling. First, an initialization step of 10 min at 50 °C was used for oil curing. Second, a 10-min “hot start” step was needed at 95 °C to activate the Taq DNA polymerase. Finally, 45 cycles of 95 °C for 20 s and 56 °C for 40 s were performed to amplify the target DNA.

### 2.4. Image Acquisition and Analysis

All bright-field images were acquired with an inverted microscope (XDS-800C, Caikon, Shanghai, China), and all fluorescence images were acquired using a Biochip reading system (Turtle Tech. Ltd., Shanghai, China). The fluorescence images were analyzed using Image J software (https://imagej.nih.gov/ij/) (accessed on 2 May 2021).

## 3. Results and Discussion

### 3.1. Reagent Self-Priming and Compartmentalization

To simplify the operation process, a self-priming microchannel dPCR chip integrated with a fractal branch and cushion chambers was developed. The principle of self-priming of this chip makes use of the high gas solubility and good air permeability of PDMS [26]. The air in the microchannel and chambers of the chip and that dissolved in the PDMS layer can be evacuated by vacuum de-gassing. A pressure difference will exist between the inside and outside of the chip when the degassed chip is brought back into the atmosphere, because the air will be quickly drawn into the inner part of the chip and re-dissolved in the PDMS slowly to reach equilibrium. In such a way, when the sample is added into the inlet of the de-gassed chip, the pressure difference will act as the self-priming power and drive the sample into the microchannel and microcavity.

**Figure 2:**
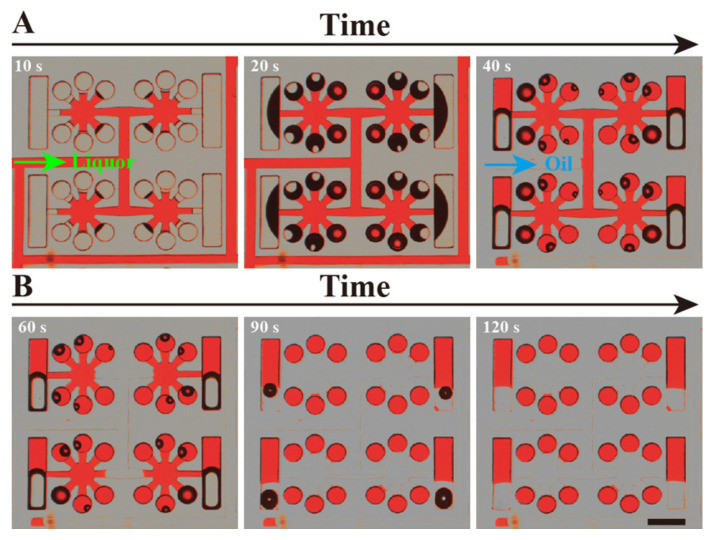
Optical micrograph of the process of red dye and oil self-primed into the dPCR chip. (**A**) The red dye solution (liquor) was sucked into the microchannel and chambers. (**B**) The oil (transparent) phase flowed into the chip and separated the red dye solution. Lastly, the oil phase entirely separates the red dye in each chamber. The black shadow in the chambers is air-water interface.

To facilitate observation, the entire process of reagent self-compartmentalization was demonstrated with red dye and oil, and is shown in Figure 2. After the dPCR chip was de-gassed in the vacuum chamber, 3.6 μL of red-dye solution and 10 μL of oil were added to the inlet successively with a two-level micro-pipette. The red dye was drawn into the microchannels and microchambers quickly by the self-priming power (Figure 2A). Approximately 20 s later, the red-dye solution was totally sucked into the chip, and gradually divided by the multi-level vertical branching microchannel structure, with the transparent oil following it (as shown in Figure 2B). Approximately 2 min later, all the red-dye solution was partitioned into each chamber by the oil, forming completely separated independent units. Figure 2B also clearly shows that all the reaction chambers are uniformly filled with red-dye solution.

### 3.2. Cushioning Effect Analysis of the Device

To achieve a homogeneous segmentation of the solution, it is not enough to depend solely on the fractal branching structure, a highly uniform pressure in the chip is also essential in different parts of the chip. A highly uniform pressure in the chip is only available if the chip has a high geometric symmetry and machining precision. Uneven negative pressure will inevitably appear in a chip without high geometric symmetry and very accurate dimensions. Chambers of the chip in an area of higher negative pressure would better take in aqueous solutions, and, on the contrary, other chambers would not take in enough reagents. As a result, part of the chambers of other multi-level vertical branching microchannel chips without cushion chambers cannot be completely separated by oil, and the other part of the chambers can also not be filled with reagent (shown in Figure S3A). The reaction chambers in the proposed chip have a lower flowing resistance compared with cushion chambers, so the reagent will be preferentially drawn into the reaction chambers. Cushion chambers can provide a buffer effect that can eliminate the failure of sample segmentation caused by uneven pressure in the chip. Figure 2B and Figure S3B clearly show that excess reagent eventually enters the cushion chambers, rather than residues in the end of the branch.

**Figure 3:**
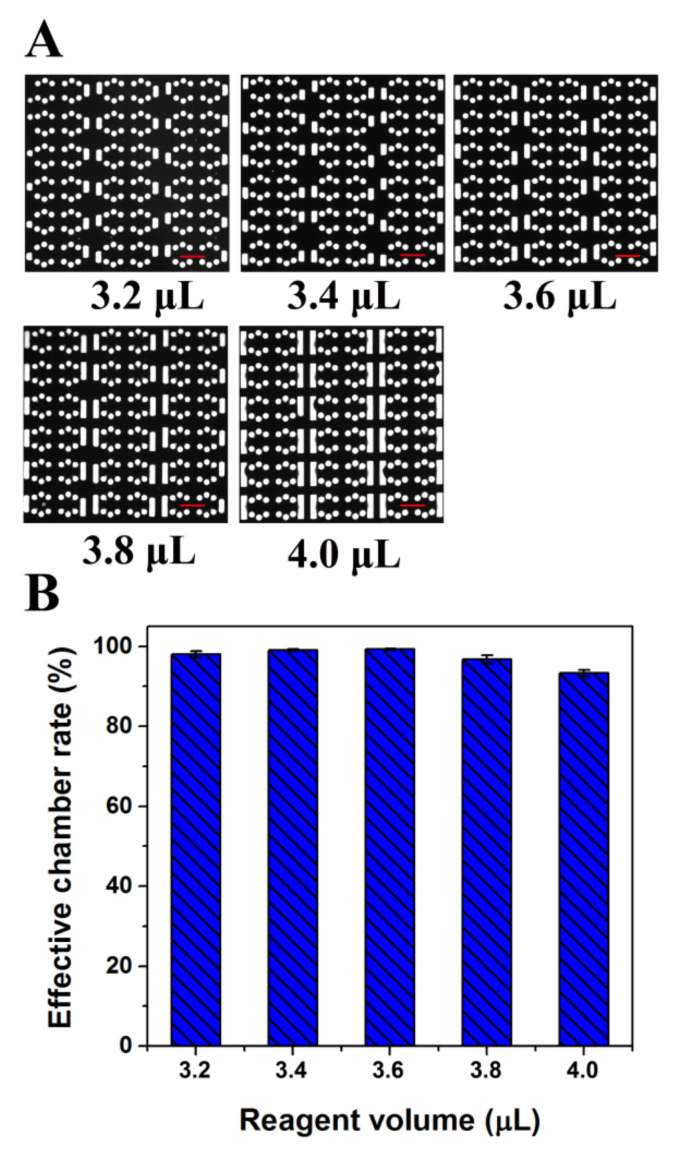
Diagram of the cushioning effect analysis of the dPCR chip. (**A**) Fluorescent image of reaction chambers filled with fluorescent solution after loading different amount of fluorescent reagent. (**B**) Effective chamber rate after loading different amount of fluorescent reagent. (All scale bars represent 400 μm).

In the same way, the use of cushion chambers can avoid the failed sample segmentation caused by operation error in pipetting. To verify the correctness of this assumption, different volumes of reagents were drawn into the chip. Figure 3A shows fluorescence microscope photographs of the chip after different volumes of reagents were dispersed. When the reagent volume used was 3.2 μL, qualified chambers (separated by oil and filled with liquid without oil and air) reached more than 98% (Figure 3B). With increasing reagent dose, the liquid drawn into the cushion chambers increased gradually. However, the qualified rate of the reaction chambers was maintained at more than 95% until the reagent volume reached 4.0 μL. Therefore, the chip has great tolerance of volume changes of the loading sample. In summary, the device has a strong buffering capacity and can completely meet the needs of robust operation, which is relatively straightforward for dPCR. To ensure the stability of sample partitioning and reduce the amount of reagent used, the reagent volume selected for further experiments was 3.4 μL. This chip may cause about a 30% liquid loss, which is less than the reagent loss on a commercial dPCR platform (around 35% liquid loss) [27,28]. Moreover, by optimizing the structure of the chip, such as by increasing the number of reaction chambers in one branch and reducing the flow resistance ratio between the reaction chamber and buffer chamber, the liquid loss could be further reduced in the proposed chip.

### 3.3. Uniformity Analysis of dPCR Chip

**Figure 4:**
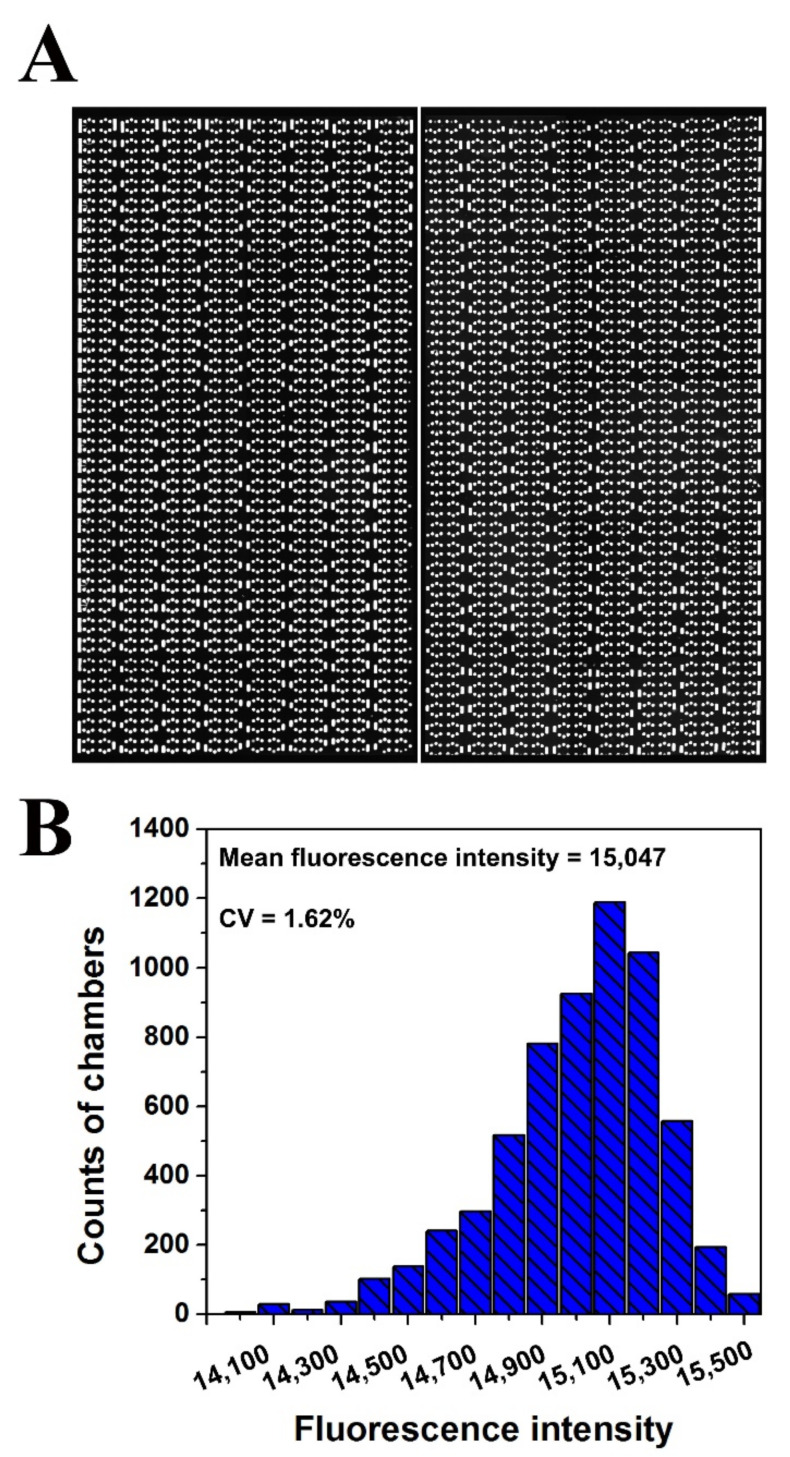
Diagram of the uniformity analysis of the dPCR chip. (**A**) Fluorescent image of reaction chambers filled with fluorescent solution. (**B**) Histogram of the fluorescence intensities distribution of all the reaction chambers (6144) in one chip.

To investigate the uniformity of the sample distributed in each reaction chamber, 3.4 μL of fluorescent dye was introduced into each panel of the chip. A fluorescence microscope photograph of the chip after sample loading is presented in Figure 4A. The fluorescence intensities of all reaction chambers (6144) of the chip were measured using Image J. Statistical analysis of the distribution of fluorescence intensity values of the reaction chambers shows that the relative standard was less than 2% (Figure 4B), indicating that the reaction chambers were filled equally and the chip had a good dispersing uniformity.

### 3.4. Absolute DNA Quantification by dPCR

**Figure 5:**
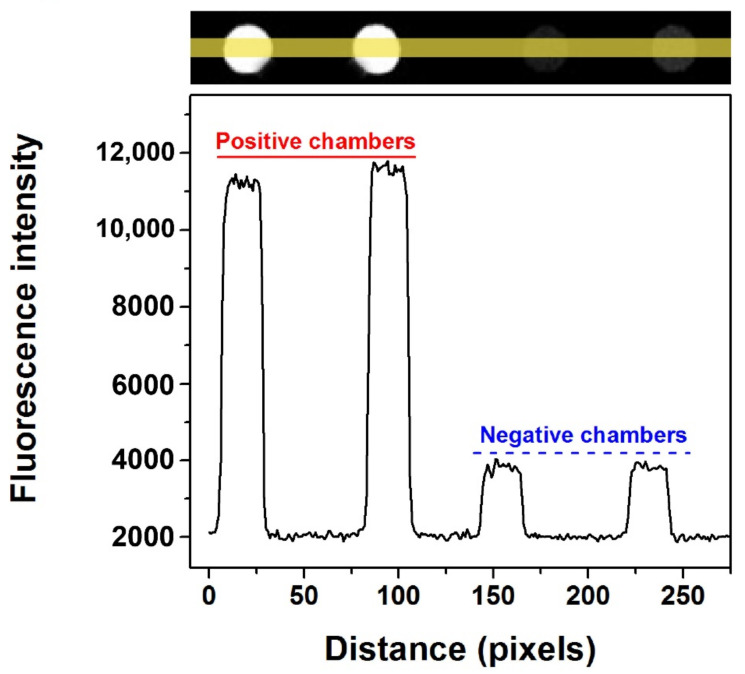
Fluorescence intensity contrast between positive and negative chambers of KRAS wild probes-CY5 with self-priming after PCR amplification. (The yellow line represents the statistical area of fluorescence intensity. The black curve below shows the change of fluorescence intensity corresponding to the chip position above).

One of the most important applications of dPCR is the absolute quantification of nucleic acid. Before conducting quantitative experiments, testing the feasibility of the microfluidic chip was necessary to perform single molecule amplification. The KRAS wild DNA template was used to perform dPCR in the self-priming chip and the CY5 fluorescence channel was selected. The fluorescent image of the proposed chip after dPCR is shown in Figure S4, where there are a few chambers with obvious water evaporation (not more than 10% in total), while most chambers in the chip did not exhibit water evaporation, which can be attributed to the glass–PDMS–glass multi-layer structure. The comparison results of the fluorescence intensity between the positive and negative chambers in the self-priming chip are shown in Figure 5, in which the bottom curve shows the change of corresponding fluorescence intensity with the change of position. It is easy to distinguish the positive chambers from the negative ones in the proposed dPCR chip because the fluorescence intensity of the positive chambers is approximately four-fold that of the negative ones for both fluorescent channels.

**Figure 6:**
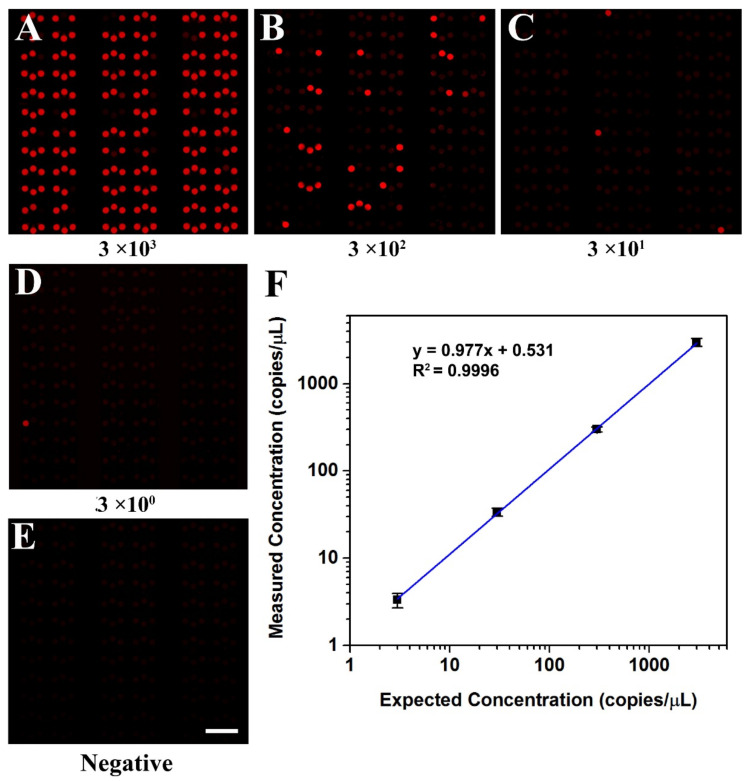
Digital PCR on the microfluidic dPCR chip with different concentrations of KRAS wild DNA template. (**A**–**D**) Digital PCR on the dPCR chip with a serial dilution of target DNA template ranging from 3 × 10^3^ copies/μL to 3 copies/μL (final concentration). (**E**) The negative assay was the control when no target template was loaded. (**F**) The linear relationship between the measured concentration in the dPCR chip and the expected DNA concentration.

To evaluate the quantitative detection ability of the self-priming microfluidic chip, the concentration of the KRAS wild DNA template was measured by a commercial digital PCR platform before performing dPCR in the self-priming chip, and its concentration was about 3 × 10^3^ copies/μL, as shown in Figure S5. Then, KRAS wild DNA templates were diluted in dilutions spanning four orders of magnitude, ranging from 3 to 3 × 10^3^ copies per μL to perform dPCR in the self-priming chip. To facilitate data analysis, the cushion chambers (not included in statistics) were removed from the fluorescence images obtained in dPCR tests, as shown in Figure S6. The fluorescence photographs of samples with different concentrations after amplification are shown in Figure 6A–D and Figure S7. The fraction of positive chambers decreased proportionally as the DNA template in the PCR master mixture was diluted, and the negative control sample was observed as no positive chambers (Figure 6E).

For digital PCR, the sample concentration is generally calculated using the Poisson distribution formula:

$$\lambda =(-ln\frac{n-b}{n})/V$$

where *n* refers to the quantity of the effective reaction chambers, *b* to the quantities of the positive chambers, *V* to the volume of reaction chambers, and λ to the calculated concentration of the experiments. Three repeated experiments were performed for each gradient.

The initial concentration of the sample can be calculated from the regression curve obtained through linear fitting (Figure 6F). It can be clearly seen from the figure that the calculated concentrations matched the expected concentrations (R^2^ = 0.9996) well. The results demonstrated that the proposed dPCR chip could calculate DNA concentration accurately and is a robust and reliable microfluidic system.

## 4. Conclusions

In this study, a multi-level vertical branching microchannel microfluidic dPCR chip with cushion chambers was proposed. The multi-level branching microchannel design enables the dPCR chip to achieve a simple method of partitioning samples. Furthermore, cushion chambers provide a simple and reliable buffering effect that effectively inhibits unfavorable sample partitions. With cushion chambers and a multi-level vertical branching microchannel, the proposed microfluidic chip can achieve sample separation with high uniformity, without an additional pump and valve. Due to its advantages of simple manufacturing processes, low production cost, and easy operation, the proposed chip is expected to further promote extensive dPCR application. Moreover, this chip has the potential to meet the requirements for an ideal diagnostic test in the developing world, owing to its low-cost, user friendliness, robustness, and resistance to cross-contamination.

## Data Availability

Not applicable.

## Supplementary Materials

The following are available online at https://www.mdpi.com/article/10.3390/bios11050158/s1, Table S1: Sequences of primers and probes used in this experiment, Figure S1: The scalability of the dPCR chip. (A) There are 24 reaction chambers in one unit, and there will be 24*n reaction chambers by folded n times; (B) There are more than 20,000 reaction chambers in the chip when n equal to 10, Figure S2: The operation flow of the self-priming dPCR chip. (A) The chip was degassed in a vacuum chamber after sealing the inlet of the chip with adhesive tape; (B) After degassing, the adhesive tape was removed, and the sample was dispensed into the inlet with a two-level micropipette, the reagent was self-primed into the chambers and separated by the oil flowing it the reagent. (C) The chip was sealed with a coverslip coated oil, Figure S3: Sample partition comparison between the existing self-priming fractal branching chip without cushion chambers (A) and the self-priming dPCR chip with cushion chambers (B), Figure S4: Whole micrograph of chip after digital PCR (A) and the local view of chip after digital PCR (B). There are a few chambers existing obvious water evaporation on the outmost arrays (shown in the yellow dotted rectangle), most chambers in the chip not existing water evaporation on other area, Figure S5: The quantitative results of KRAS templates by a commercial digital PCR platform. (The concentration of KRAS templates measured is 3266.9 ± 77.5 copies/μL, repeated three times.), Figure S6: Original image (A) and cushion chambers removed image (B) after dPCR on the chip. The scale bar is 200 μm, Figure S7: Whole micrographs for each micrograph of chip after digital PCR.
