# Supplementary material for: A Self-Priming Microfluidic Chip with Cushion Chambers for Easy Digital PCR
Source: Biosensors (Basel). 2021 May 18;11(5):158. doi: 10.3390/bios11050158 (PMC8155915; doi:10.3390/bios11050158)
Supplement: Supplementary file 1 [file biosensors-11-00158-s001.zip › biosensors-1171182-supplementary.pdf]

Supporting Information

A Self-Priming Microfluidic Chip with Cushion Chambers for Easy Digital PCR

Gangwei Xu [1] , Huaqing Si [1], Fengxiang Jing [2], Peng Sun [1] and Dongping Wu [1,*]

[1] State Key Laboratory of ASIC and System, School of Microelectronics, Fudan University, Shanghai 200433, China; 18112020050@fudan.edu.cn (G.X.); 17112020035@fudan.edu.cn (H.S.); 18112020068@fudan.edu.cn (P.S.)

[2] Shanghai Turtle Technology Company Limited, Shanghai, 200439, China; fengxiangjing@turtle-tech.cn

* Correspondence: dongpingwu@fudan.edu.cn

Note 1. Fabrication process of the silicon mold

Standard multilayer soft lithography techniques were used for fabricating the microfluidic chip as our previous work [1]. Two-layer separate photoresist masks for microchannels and microchambers were designed using computer-aided design software Auto CAD (https://www.autodesk.com.cn/) and printed on transparency films. A 4-inch silicon wafer was washed ordinal with acetone, alcohol and deionized water by ultrasonic cleaning, and then baked at 200 °C for 10 min. Then a 30 μm thick layer of an SU-8 3050 negative photoresists (MicroChem Co., Ltd, USA) was spun on the cleaned silicon wafer at 3000 rpm for 30 s for making microchannels. The coated silicon wafer was exposed to ultraviolet radiation using a mask aligner (NXQ4006 Mask Aligner, California, USA). After exposure and development, the silicon wafer was hard baked on a hot plate at 200 °C for 30 min. Next, a 100 μm thick layer of an SU-8 3050 negative photoresists were used to construct the microchambers. After the features of the microchambers on the mask were aligned with the flow channels, the photoresist was exposed, baked and developed, until the mold was finished. Lastly, the mold was hard baked at 200 °C for 1 hour. The chip was mainly made of PDMS. First, premixed 10: 1(part A: part B) PDMS (Dow Corning, USA) was mixed with Triton X-100 (Sigma Aldrich, St Louis, USA) at 0.1% weight percentage. Subsequently, the mixture was degassed in vacuum, then injected over the master using a syringe and cured at 100 °C for 30 min. After being cured, the PDMS was peeled off the master and punched hold for inlet. Lastly, the chip was bonded on a clean glass coverslip by plasma treatment. The assembled device was then baked at 120 °C for 1 hours before use.

Table S1. Sequences of primers and probes used in this experiment.

Forward primer	5'-CCTGCTGAAAATGACTGAA-3'
Reverse primer	5'-AAGATTTACCTCTATTGTTGG-3'
KRAS Reference probe	5'-CY5-AGAGTGCCTTGACGATACAGCTAA-AF594-3'

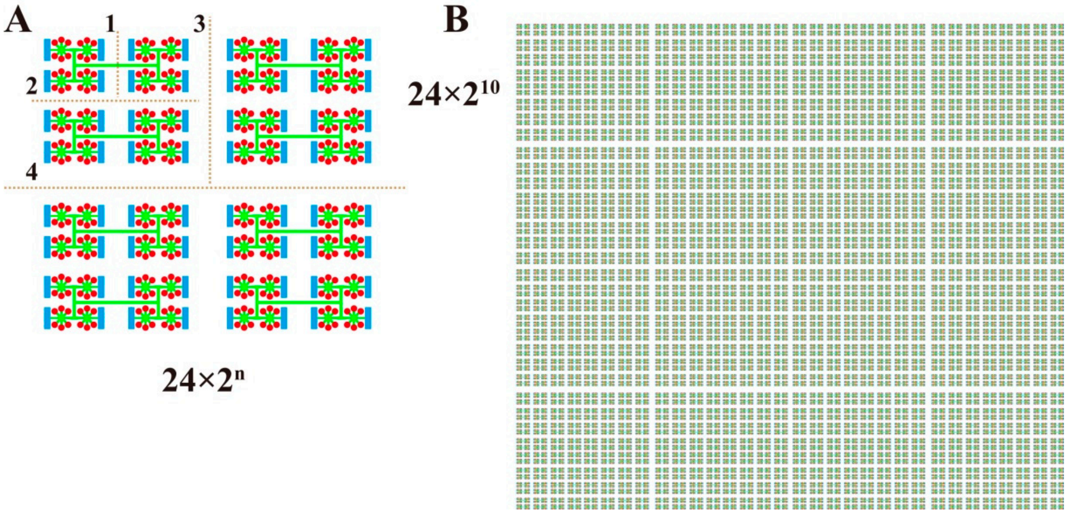

Figure S1. The scalability of the dPCR chip. (A) There are 24 reaction chambers in one unit, and there will be 24*n reaction chambers by folded n times; (B) There are more than 20,000 reaction chambers in the chip when n equal to 10.

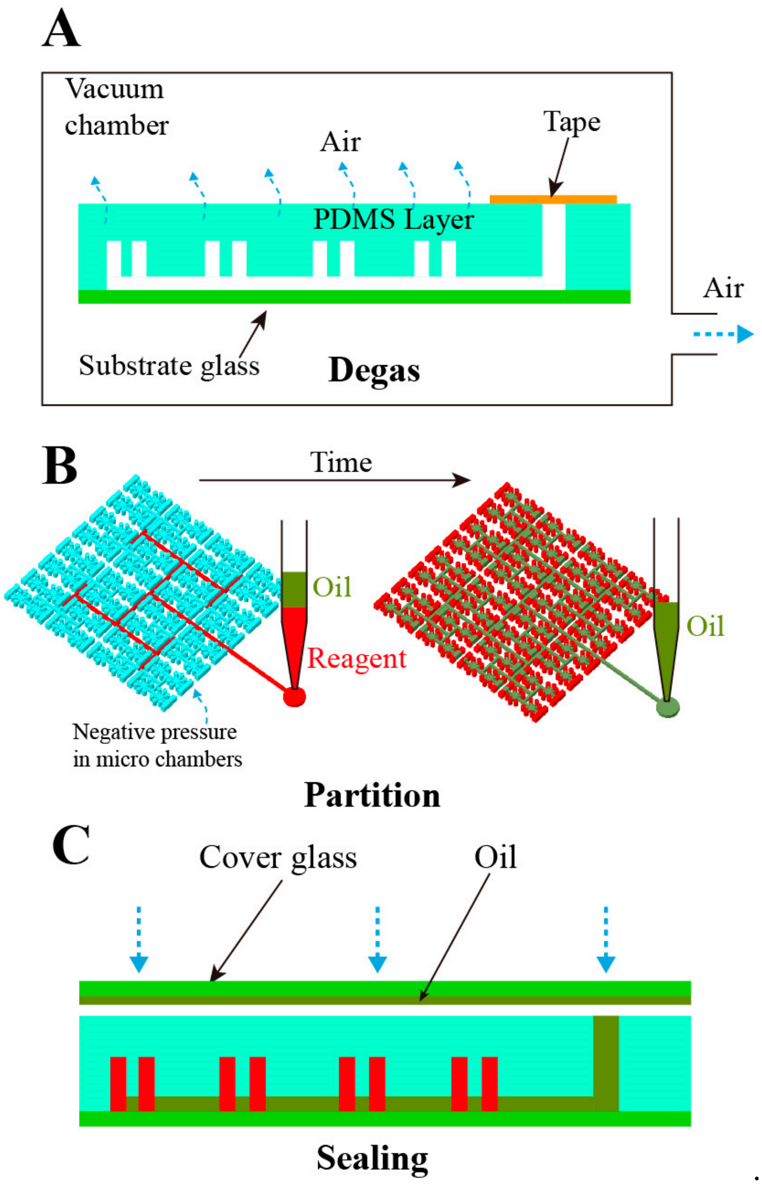

Figure S2. The operation flow of the self-priming dPCR chip. (A) The chip was degassed in a vacuum chamber after sealing the inlet of the chip with adhesive tape; (B) After degassing, the adhesive tape was removed, and the sample was dispensed into the inlet with a two-level micropipette, the reagent was self-primed into the chambers and separated by the oil flowing it the reagent. (C) The chip was sealed with a coverslip coated oil.

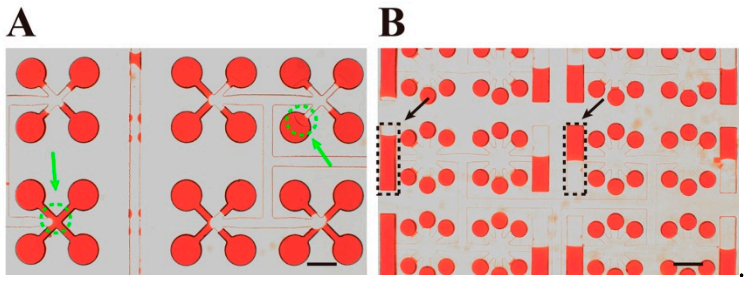

Figure S3. Sample partition comparison between the existing self-priming fractal branching chip without cushion chambers (A) and the self-priming dPCR chip with cushion chambers (B).

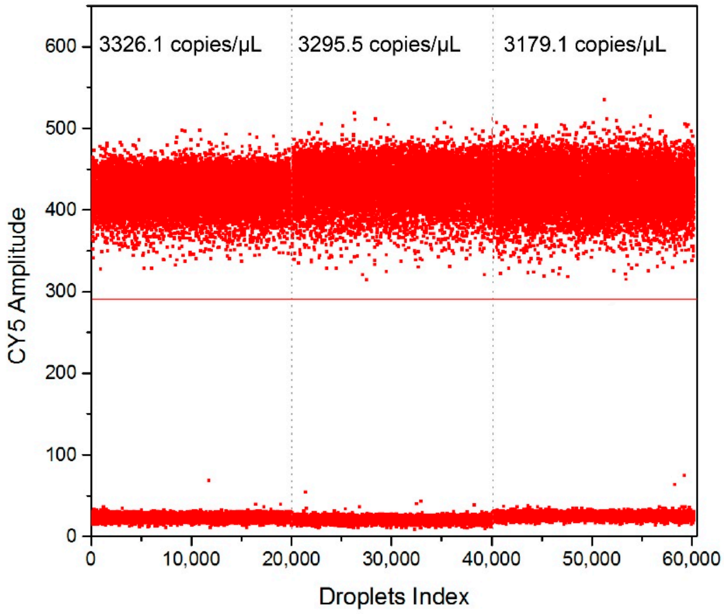

Figure S4. The quantitative results of KRAS templates by a commercial digital PCR platform. (The concentration of KRAS templates measured is 3266.9±77.5 copies/μL, repeated three times.).

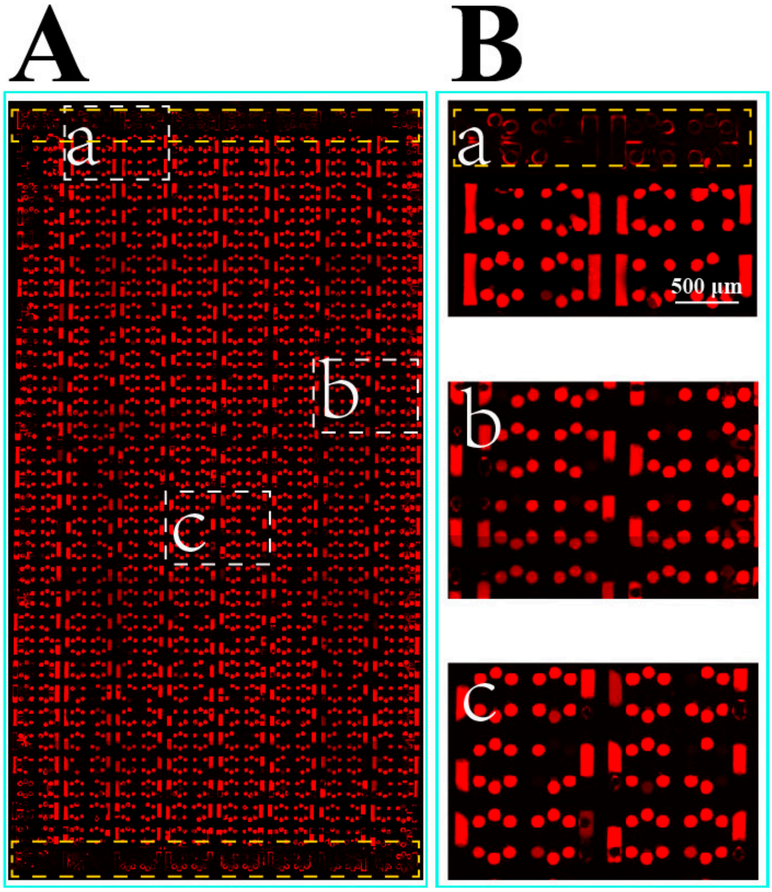

Figure S5. Whole micrograph of chip after digital PCR (A) and the local view of chip after digital PCR (B). There are a few chambers existing obvious water evaporation on the outmost arrays (shown in the yellow dotted rectangle), most chambers in the chip not existing water evaporation on other area.

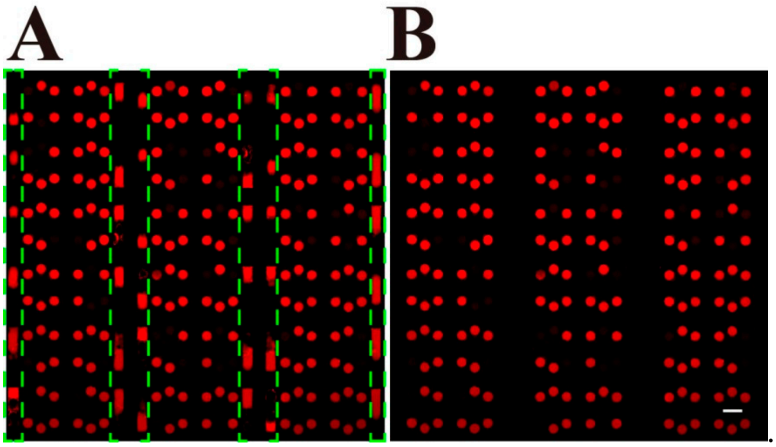

Figure S6. Original image (A) and cushion chambers removed image (B) after dPCR on the chip. The scale bar is 200 μm.

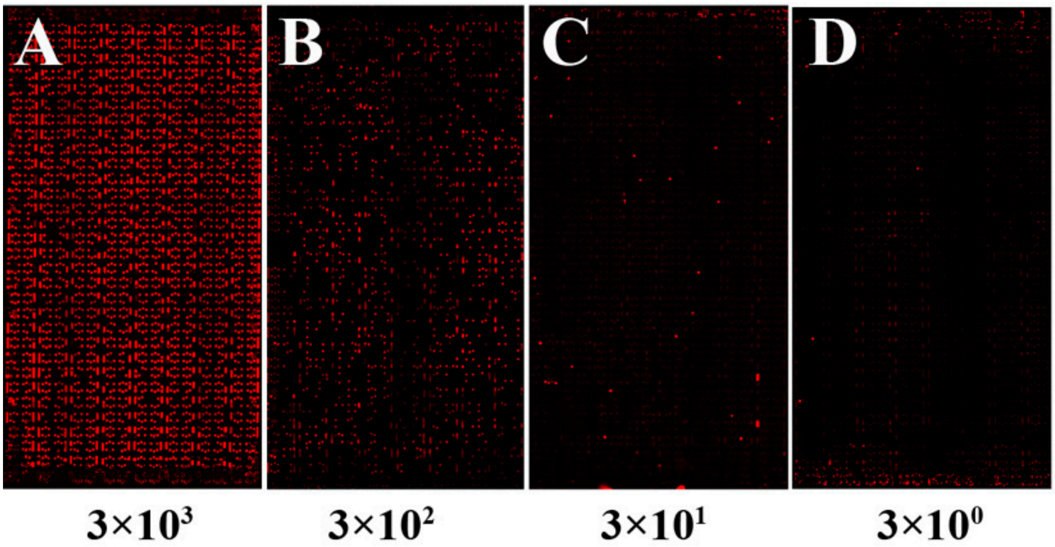

Figure S7. Whole micrographs for each micrograph of chip after digital PCR.

References

1. [1] Si, H.; Xu, G.; Jing, F.; Sun, P.; Zhao, D.; Wu, D. A multi-volume microfluidic device with no reagent loss for low-cost digital PCR application. *Sens. Actuators B Chem.* **2020**, *318*, 128197.
